# Fascia Wrapping Technique: A Modified Method for the Treatment of Cubital Tunnel Syndrome

**DOI:** 10.1155/2014/482702

**Published:** 2014-10-15

**Authors:** Hyun Ho Han, Hae Won Kang, Jun Yong Lee, Sung-No Jung

**Affiliations:** Department of Plastic and Reconstructive Surgery, Uijeongbu St. Mary's Hospital, College of Medicine, The Catholic University of Korea, 222 Banpo-daero, Seocho-gu, Seoul 137-701, Republic of Korea

## Abstract

Variations of the anterior transposition of the ulnar nerve for cubital tunnel syndrome include subcutaneous, submuscular, intramuscular, and subfascial methods. We introduce a modification of subfascial transposition, which is designed to facilitate nerve gliding by wrapping the nerve with fascia. Twenty patients with wrapping surgery following the diagnosis of cubital tunnel syndrome were reviewed retrospectively. Preoperative electrodiagnostic studies were performed in all patients and all of them were rechecked postoperatively. The preoperative mean value of motor conduction velocity (MCV) was 37.1 ± 6.7 m/s within the elbow segment and this result showed a decrease compared to the result of MCV with 53.9 ± 6.9 m/s in the below the elbow-wrist segment with statistical significance (*P* < 0.05). Postoperative mean values of MCV were improved in all of 20 patients to 47.6 ± 5.5 m/s (*P* < 0.05). 19 patients of 20 (95%) reported good or excellent clinical outcomes according to a modified Bishop scoring system. The surgical treatment methods for cubital tunnel syndrome have their own advantages and disadvantages, and the preferred method differs depending on the surgeon. The wrapping method of anterior transposition is a newly designed alternative method modified from subfascial transposition. This method could be an alternative option to treat cubital tunnel syndrome.

## 1. Introductions

Ulnar nerve compression at the elbow region, which is named cubital tunnel syndrome, is the second most common compressive neuropathy of the upper limb after carpal tunnel syndrome [1]. Multiple surgical options have been recommended in the literature and reflect the controversy surrounding the surgical treatment of cubital tunnel syndrome. The surgical management is broadly divided into three types of procedures [2]: simple decompression [3, 4], medial epicondylectomy [5, 6], and anterior transposition of the ulnar nerve. Also variations of anterior transposition of the ulnar nerve have been proposed; these include subcutaneous [7–9], submuscular [10–12], intramuscular  [13–15], and subfascial [2, 16] methods. A subcutaneous transposition is a simple and reliable procedure that facilitates an early postoperative mobilization. However, it is more vulnerable to trauma and hypersensitivity. A submuscular or intramuscular transposition is well protected as it lies deeply under a substantial amount of soft tissue. However, it has the disadvantages of prolonged postoperative elbow immobilization and potential subsequent contracture. A subfascial transposition protects the transposed nerve and avoids problems like scarring, recurrence, and elbow contracture [2, 16].

The method we are introducing in this study is a modified method of subfascial transposition. The method of subfascial transposition has merits as mentioned above, but whether the nerve would not adhere between the fascia and the muscle and gliding would be facilitated was doubted, and there was a difficulty in fixing the fascial flap to the muscle after elevation since the muscular tissue was friable [17, 18]. In addition, if there is a defect on a region like dorsum of hand for which tendon gliding is necessary, to cover it with temporoparietal fascia free flap to facilitate tendon gliding after coverage and conduct split thickness skin graft is a widely known method [19–21] based on which we thought that wrapping the nerve with fascia would cause less adhesion and be helpful for gliding. Thus this new method is designed to facilitate nerve gliding by wrapping the nerve with fascia. Here, we summarize and report the surgery method as well as the result of surgery.

## 2. Patients and Method

Twenty patients who had surgery with the wrapping method due to the diagnosis of cubital tunnel syndrome were reviewed retrospectively. The study patient pool consists of patients who had a surgical operation at a single centre in Uijeongbu St. Mary's Hospital from January 2008 through January 2012. All operations have been conducted by the corresponding author, Sung-No Jung. Diagnosis of cubital tunnel syndrome was made on a typical history of pain. Sensory deficit according to the distribution of ulnar nerve was measured by static and dynamic 2-point discrimination tests. And the loss of intrinsic bulk and weakness of grip strength were measured as well using a hand grip dynamometer and compared with that of the normal part on the opposite side. Preoperatively, the condition of the ulnar nerve was graded according to severity, based on Dellon's classification [22]. Preoperative electromyography of the flexor carpi ulnaris, abductor digiti minimi, and first interosseous muscle was done in all patients. Also we evaluated the preoperative motor conduction velocity (MCV) of the ulnar nerve in the segments of below the elbow-wrist, above the elbow-below the elbow, and axilla-above the elbow in all patients. A section survey was simultaneously applied between 4 cm distal and 6 cm proximal to the medical epicondyle in all patients to determine the exact location of the compression site. Patients were selected for the operation by using the following 2 criteria: (1) an absolute MCV from above the elbow to below the elbow of less than 50 m/s or (2) slowing of greater than 10 m/s in the above the elbow to below the elbow segment compared with the below the elbow to wrist segment [23]. Also differential diagnosis was conducted for polyneuropathy. All patients were preoperatively examined with standard radiographs of the elbow.

The fascia wrapping method was used for the surgery in all patients. The postoperative MCV test and outcome assessment for the patients were based on the modified Bishop scoring system [14]. It was examined in all 20 patients about 1 year later.

The MCV results between preoperative and postoperative data were statistically analyzed by independent *t*-test and the results between preoperative below the elbow-wrist and within the elbow segment were analyzed by one sample *t*-test. SPSS version 13.0 software (SPSS Inc., Chicago, IL, USA) was used.

### 2.1. Operative Procedure

**Figure 1:**
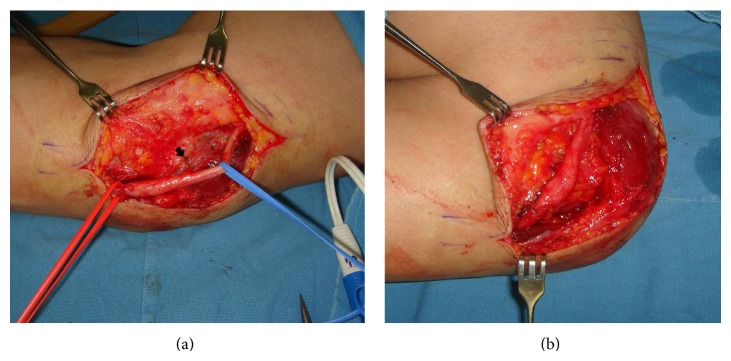
Nerve release and anterior transposition. (a) Feeding artery vessel (arrow) of nerve must be saved. (b) Anterior transposition of the ulnar nerve was conducted followed by dissection to achieve sufficient release without compression of the ulnar nerve.

**Figure 2:**
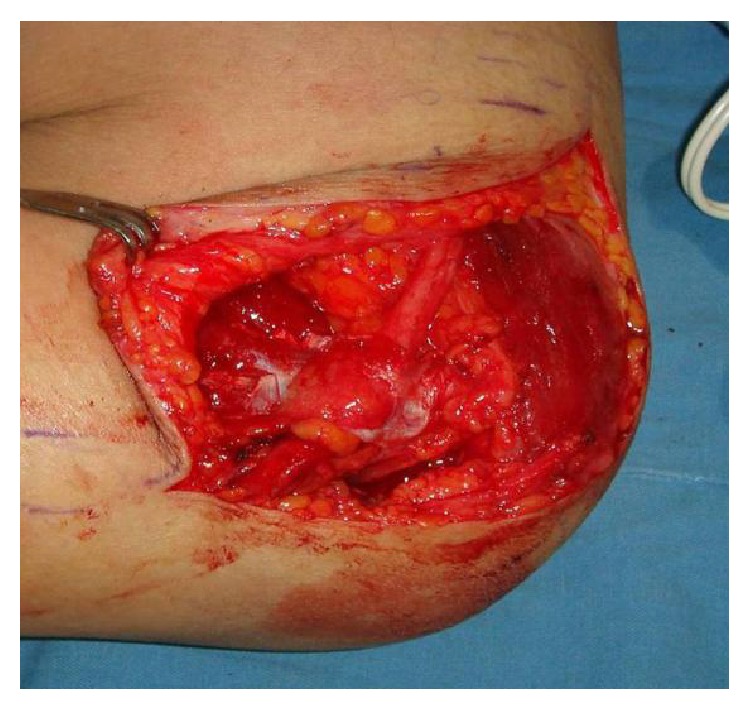
Wrapping procedure. Wrapping procedure was conducted by locating the ulnar nerve over the fascia and very loosely rolling the ulnar nerve with the elevated fascia flap. Closure could be tightly made together with the fascia.

**Figure 3:**
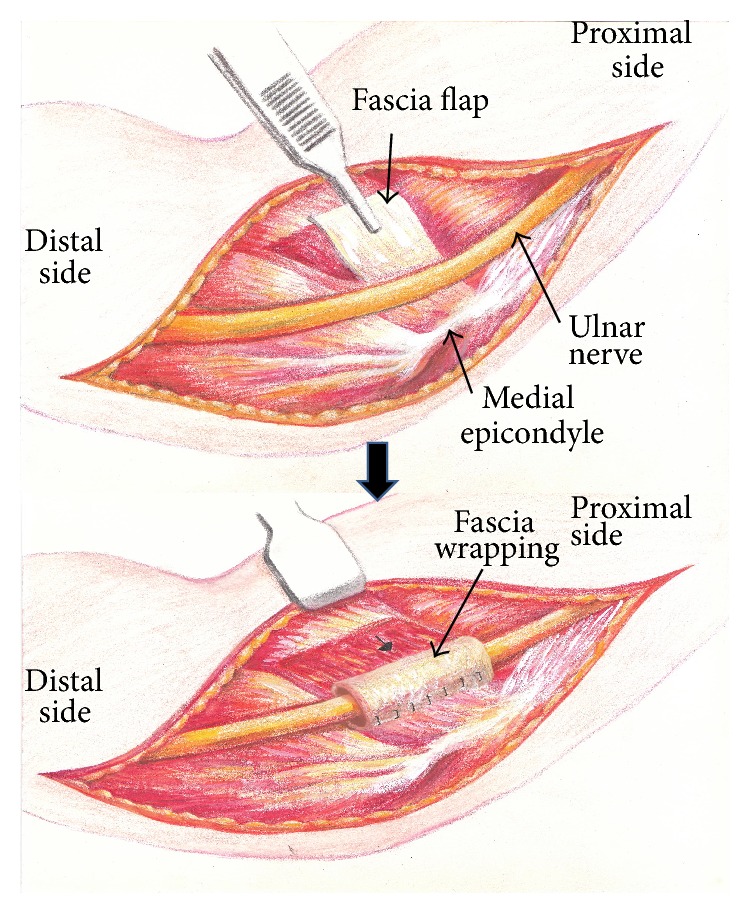
Schematic illustration of the wrapping procedures.

Skin and underlying subcutaneous tissues were curvilinear incised midway between the olecranon and medial epicondyle under general anesthesia and tourniquet control. The fascia was divided between the medial epicondyle and olecranon, passing proximally from the medial intermuscular septum to the postcondylar groove, releasing Osborne's band, which is an aponeurosis located between the two heads of flexor carpi ulnaris muscle. The feeding artery vessel of the nerve should be saved (Figure 1). An anterior transposition of the ulnar nerve was conducted followed by dissection to achieve a sufficient release without compression of the nerve (Figure 1). Superficial fascia belonging to the flexor pronator muscle group was elevated as a broad fascia flap with a width exceeding approximately 3 cm and a position of 1-2 cm apart from the medical epicondyle origin. Unlike the existing subfascial transposition, which is located in the nerve between muscle and fascia after elevating fascia flap, we conducted the wrapping procedure by locating the ulnar nerve over the fascia and very loosely rolling the ulnar nerve with the elevated fascia flap (Figures 2 and 3). The elevated fascia flap was firmly anchored onto the fascia located to the side of medial epicondyle through a continuous absorbable suture. The course of the transposed ulnar nerve was then rechecked to ensure that there was no kinking or compression. No drains were inserted for all patients.

### 2.2. Postoperative Care and Rehabilitation

After the surgery was the splint maintained for 3-4 days along with moderate compressive dressing. After this time, the splint was removed and exercise commenced with gentle flexion and extension of the elbow after the swelling subsided. Full activities were possible within 4 weeks. A regular follow-up every 3–6 months was essential.

## 3. Results

**Figure 4:**
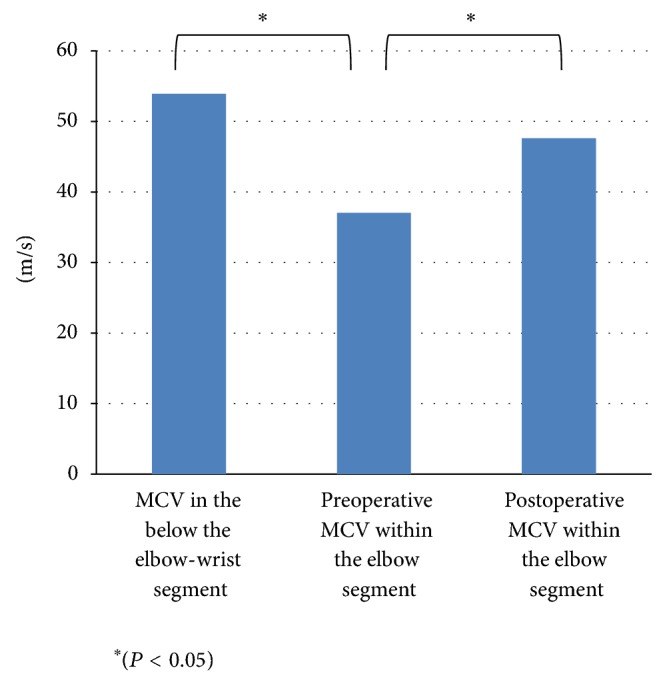
Motor conduction velocity (MCV) result. The preoperative mean value of motor conduction velocity (MCV) was 37.1 ± 6.7 m/s within the segment (above the elbow-below the elbow) and this result showed a decrease compared to the result of MCV with 53.9 ± 6.9 m/s in the below the elbow-wrist segment with statistical significance (*P* < 0.05). Postoperative mean values of MCV were improved in all of 20 patients to 47.6 ± 5.5 m/s (*P* < 0.05).

Twenty patients (15 males, 5 females; average age 49 years, range 33–68 years) were studied. Patient details are summarized in Table 1. Eight (40%) patients had a medical history with preoperative injury. Sensory reduction was evident in all 20 patients. 11 (55%) patients demonstrated intrinsic atrophy, grip strength was reduced in 17 (85%) patients, and 17 (85%) patients were positive for Tinel's sign. Dellon's classification was conducted to evaluate all of the preoperative patients and 7 out of 20 patients were graded as III (severe syndrome), 11 were graded as II (moderate syndrome), and 2 were graded as I (mild syndrome) (Table 2). Preoperative electrodiagnostic abnormalities were seen in all 20 patients elbows which underwent MCV examinations across the elbow segment of the ulnar nerve. The mean value of MCV within the segment was 37.1 ± 6.7 m/s and it was (53.9 ± 6.9 m/s) more decreased than the value of MCV in the below the elbow-wrist segment of the involved limbs with statistically significant difference (*P* < 0.05) (Figure 4).

The average follow-up period was 24 months (ranging from 9 to 32 months) and a postoperative electrophysiological study was assessed about one year after the surgery in all 20 patients. The mean value of MCV had improved from 37.1 ± 6.7 m/s to 47.6 ± 5.5 m/s (*P* < 0.05) with statistical significance. Subjective symptoms were also improved in all patients one year after the surgery. 19 patients of 20 (95%) reported good or excellent clinical outcomes according to a modified Bishop scoring system (Table 3). There were no complications, recurrence, or subluxation of the ulnar nerve.

## 4. Discussion

There are many different opinions about pathogenesis, surgery methods, and results for the patients diagnosed with cubital tunnel syndrome. Hence, the surgical approach can vary depending on the surgeon's preference [24–26].

The size of the cubital tunnel is reduced when there is flexion of the elbow; the volume in general is reduced by 55% and pressure on the ulnar nerve increases compared to when the elbow is extended [27–29]. This can cause ischemic damage on the nerve as the length of the nerve is extended by approximately 4–7 mm, leading to a traction state [30]. Nerve compression and traction can cause microcirculatory disturbance and inflammation in the ulnar nerve, which ultimately reduces the function of the nerve [31]. In this respect, the anterior transposition method that moves the ulnar nerve from the retrocondylar position is effective in preventing compression and allowing release of nerve tension.

Previously described criteria were used to analyze the indication of surgery: (1) an absolute MCV from above the elbow to below the elbow of less than 50 m/s or (2) slowing of greater than 10 m/s in above the elbow to below the elbow segment compared with the below the elbow to wrist segment [23]. No surgery was conducted in the cases caused by another region, such as compressive ulnar neuropathy, cervical radiculopathy, thoracic outlet syndrome or Guyon's canal syndrome, and presence of angular deformity in the elbow, as well as in the cases with noncompressive neuropathy caused by diabetes mellitus, chronic renal failure, and hypothyroidism.

The wrapping method is a modification of the subfascial method, which bestows nearly the same benefit, because this technique preserves the subfascial method. The most important advantages obtained with subfascial method are less scarring and fast recovery due to a small area of dissection site compared to the submuscular and intramuscular methods [2, 16].

However, the wrapping method has more other benefits. The wrapping method has much less dissection area than the subfascia method and the surgery technique is simple because it anchors the fascia together that is not friable. In the classical subfascia method, the nerve is positioned on the subfascial plane, which is made as a separation occurring between the fascia and muscles. On the contrary, in the wrapping method, the nerve is placed on the intact, healthy, and nontraumatic anatomical fascia surface by elevating and wrapping the nerve with noninjured fascia. Therefore, the nerve adheres less and glides easily because it runs inside the healthy and nontraumatic fascia surface. For these reasons, we could assume that this method will be a great help to improve symptoms quickly and prevent recurrence by minimizing the possibility of an additional injury on the nerve of the surgery site after surgery.

Kokkalis et al. [32] also reported wrapping method using the saphenous vein which is similar concept to our new method. With the intima of the saphenous vein against the ulnar nerve, the vein is circumferentially wrapped from distal part to proximal part around the exposed nerve. They assumed that autologous vein graft with its smooth inner surface should improve the gliding function of the nerve and reduce scar formation around the nerve.

A further advantage for this surgical method is that the nerve can be placed in a more superficial position than the fascia. It is less likely that there will be problems of nerve kinking or iatrogenic compression on the new surgery site because the nerve plane is placed on the same plane before surgery, above the fascia. In the classical subfascial transposition method, there is the potential that compression on a certain region between the two heads of the FCU, which is mainly the distal part, can developed, or compression can worsen because the plane is changed from suprafascia to subfascia. For this reason, the confirmation and release of six anatomic compression sites of ulnar nerve should be completed when an anterior transposition is conducted [33]. By our wrapping method, the nerve is pre- and postoperatively moved on the same plane so the compression potential can be reduced.

Finally, the last benefit of this surgical method is that it is simple to fix the fascia. Fascia Z-plasty or step ladder incision that sutures the fascia together is used for conduction of subfascia nerve transposition due to the high potential of loosening when fascia is fixed onto muscle that is friable after nerve transposition [17, 18]. In this situation, a dissection has to be done more on the radial side. On the contrary, the wrapping method can provide simple and firm immobilization by elevating the fascia only, placing the nerve on the fascia, turning over the elevated fascia, and finally suturing the fascia together.

The limitation of our study is that we were not able to objectively analyze the surgery results of the wrapping method compared with the classical subfascia method by a control group. This study has left much to be desired and, therefore, we are looking forward to a comparative analysis between the wrapping method and subfascia method to achieve more interesting and significant results.

## 5. Conclusions

The surgical treatment methods for cubital tunnel syndrome have their own advantages and disadvantages, and the preferred method differs depending on the surgeon. The wrapping method of anterior transposition as reported in this study is a newly designed alternative method modified from subfascial transposition. This method provides better immobilization and requires less dissection than a subfascial transposition. This method could be an alternative option to treat cubital tunnel syndrome.

## Figures and Tables

**Table 1:** Patient data.

Total patient number	20 patients (M: 15 and F: 5)
Average age	49 years (range: 33–68)
History of trauma	8 patients (40%)
Sensory decrease	20 patients (100%)
Intrinsic atrophy	11 patients (55%)
Tinel's sign	17 patients (85%)
Weakness of grip strength	17 patients (85%)
Abnormal motor nerve conduction velocity (<50 m/s)	20 patients (100%)

**Table 2:** Dellon's classification.

	Mild (I)	Moderate (II)	Severe (III)
Sensory	Intermittent paresthesia	Intermittent paresthesia	Permanent paresthesia
Motor	Measurable weakness	Measurable weakness	Palsy
Patients in this study	2 (10%)	11 (55%)	7 (35%)

**Table 3:** A modified Bishop scoring system.

	Dellon I (*n* = 2)	Dellon II (*n* = 11)	Dellon III (*n* = 7)	All
Bishop-rate				
Excellent	2	7	2	11 (55%)
Good	0	4	4	8 (40%)
Fair	0	0	1	1 (5%)
Poor	0	0	0	0
